# P-665. Etiology of Pneumonia and Antibiotic Resistance in Adults with Cancer in the ICU of a Tertiary Care Centre in Eastern India: a Prospective Cohort Study

**DOI:** 10.1093/ofid/ofae631.862

**Published:** 2025-01-29

**Authors:** Simran Malik, Sanjay Bhattacharya, Sangeeta Das Bhattacharya

**Affiliations:** Indian Institute of Technology Kharagpur, Kharagpur, West Bengal, India; Tata medical center kolkata, Kolkata, West Bengal, India; Indian Institute of Technology Kharagpur, Kharagpur, West Bengal, India

## Abstract

**Background:**

Cancer and its treatment cause immune-dysregulation which, along with frequent healthcare exposure, poses a higher risk of respiratory infections, frequently with unusual pathogens that can be highly drug-resistant. We assessed the etiology of pneumonia in adult cancer patients admitted to the ICU over one year and summarized the antibiotic resistance seen.

**Figure:**
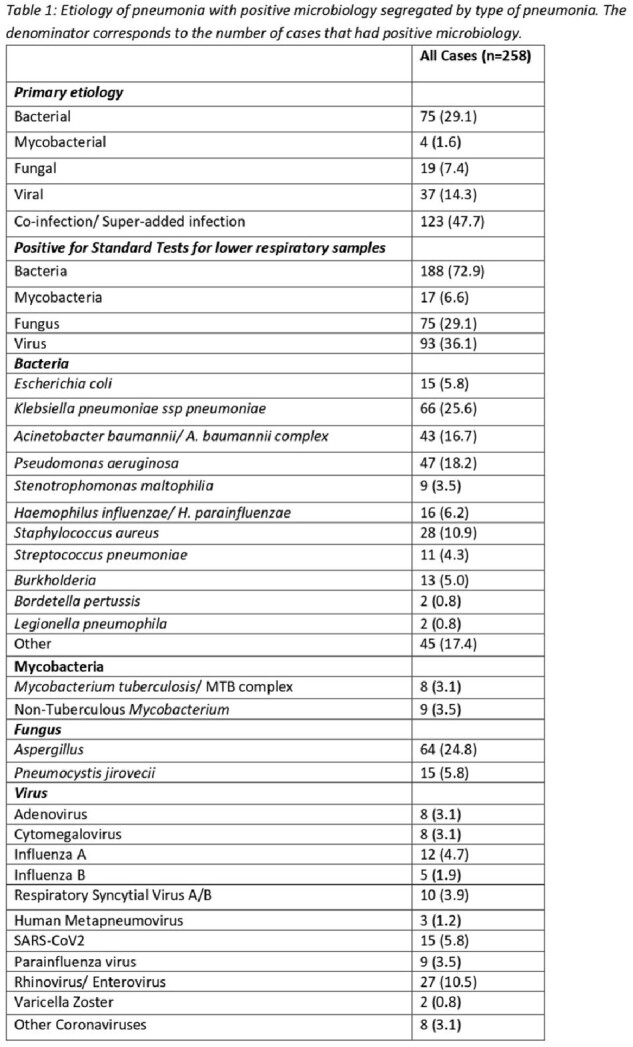

**Methods:**

Pneumonia cases were identified from October 2022-September 2023. Sputum, endotracheal secretion, bronchoalveolar lavage (BAL), tracheal aspirate, nose and throat swab samples from -4 to +10 days of the date of event were considered. Microbiological diagnosis involved microscopy, culture, ELISA, PCRs, and CBNAATs. Antibiotic susceptibility was determined phenotypically by disc-diffusion and genotypically using PCR tests for MRSA, ESBL, carbapenemase, and polymyxin resistance genes.

**Figure:**
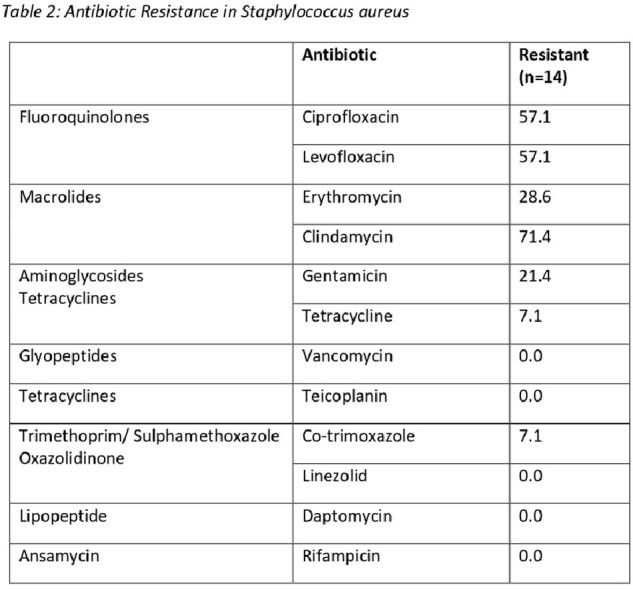

**Results:**

355 cases of pneumonia were identified. 258 (72.7%) cases of had a positive microbiology, 123 were polymicrobial (47.7%) and termed “co-infections/ superadded infections". Table 1 summarizes the etiology of microbiology positive cases.

Antibiotic susceptibility data was available for 191 isolates, 14 were *Staphylococcus aureus* (table 2) and 177 were Gram-negatives (table 3). *S. aureus* have fluoroquinolones. 30.4% were MRSA and 21.4% showed inducible clindamycin resistance.

Gram negatives were resistant to third-generation cephalosporins, beta-lactam/beta-lactamase inhibitors, carbapenems, aminoglycosides, fluoroquinolones and cotrimoxazole, though variations exist across genera. 51% Gram negative isolates were positive for ESBL, 5.8% for AmpC, 9.7% were polymyxin resistant, and 49.5% were resistant to carbapenems. Carbapenem resistance genotypes are summarized in table 4.

**Figure:**
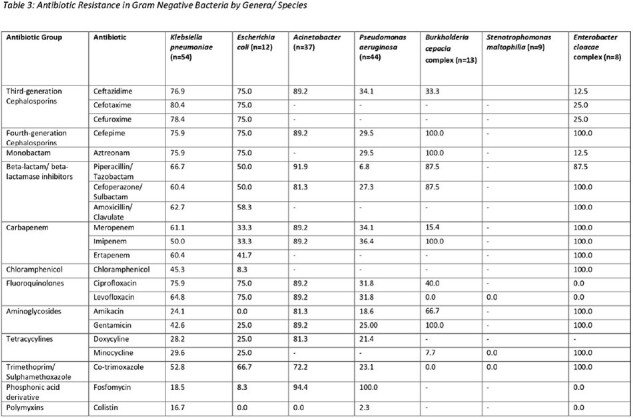

**Conclusion:**

Isolates from adult cancer patients with pneumonia showed high prevalence of resistance to all first-line antibiotics.

**Figure:**
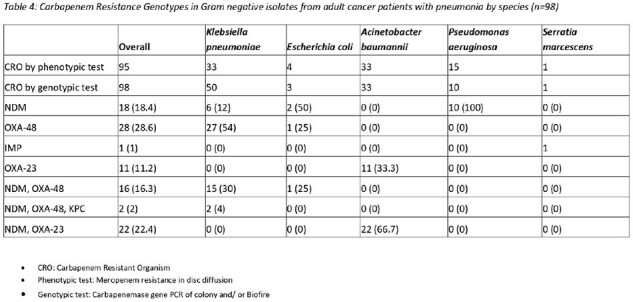

**Disclosures:**

**All Authors**: No reported disclosures

